# Mid-Atlantic Big Brown and Eastern Red Bats: Relationships between Acoustic Activity and Reproductive Phenology

**DOI:** 10.3390/d14050319

**Published:** 2022-04-21

**Authors:** Sabrina Deeley, W. Mark Ford, Nicholas J. Kalen, Samuel R. Freeze, Michael St. Germain, Michael Muthersbaugh, Elaine Barr, Andrew Kniowski, Alexander Silvis, Jesse De La Cruz

**Affiliations:** 1 Environmental Protection Agency, 1200 Pennsylvania Ave, Washington, DC 20460, USA; 2 U.S. Geological Survey, Virginia Cooperative Fish and Wildlife Research Unit, Blacksburg, VA 24061, USA; wmford@vt.edu; 3 Conservation Management Institute, Virginia Tech, Blacksburg, VA 24061, USA; nkalen@vt.edu (N.J.K.); mstgerma@vt.edu (M.S.G.); delacruz@vt.edu (J.D.L.C.); 4 Department of Fish and Wildlife Conservation, Virginia Tech, Blacksburg, VA 24061, USA; srfreeze@vt.edu (S.R.F.); kniowski@vt.edu (A.K.); 5 Department of Forestry and Environmental Conservation, Clemson University, Clemson, SC 29634, USA; mmuther@g.clemson.edu; 6 U.S. Fish and Wildlife Service, Ohio River Islands National Wildlife Refuge, Williamstown, WV 26187, USA; elaine_barr@fws.gov; 7 West Virginia Division of Natural Resources, Elkins, WV 26241, USA; alexander.silvis@wv.gov

**Keywords:** acoustic sampling, bats, big brown bats, eastern red bats, *Eptesicus fuscus*, *Lasiurus borealis*, mid-Atlantic, migration, reproduction

## Abstract

Acoustic data are often used to describe bat activity, including habitat use within the summer reproductive period. These data inform management activities that potentially impact bats, currently a taxa of high conservation concern. To understand the relationship between acoustic and reproductive timing, we sampled big brown bats (*Eptesicus fuscus*) and eastern red bats (*Lasiurus borealis*) on 482 mist-netting and 35,410 passive acoustic sampling nights within the District of Columbia, Maryland, Pennsylvania, Virginia, and West Virginia, 2015–2018. We documented the proportion of female, pregnant, lactating, and juvenile big brown and eastern red bats within each mist-net sampling event and calculated locally estimated non-parametric scatterplot smoothing (LOESS) lines for each reproductive and acoustic dataset. We compared the peak in acoustic activity with the peaks of each reproductive condition. We determined that the highest levels of acoustic activity within the maternity season were most associated with the period wherein we captured the highest proportions of lactating bats, not juvenile bats, as often assumed.

## 1. Introduction

Many North American bat species migrate from overwintering sites to summer habitat in the spring, where females give birth to offspring. They then begin returning to overwintering sites in late summer and early fall [1,2]. Big brown bats (*Eptesicus fuscus*) hibernate in caves or anthropogenic structures, and in the summer, groups of <100 females form maternity colonies usually in tree cavities or anthropogenic structures [3,4,5]. Eastern red bats (*Lasiurus borealis*) typically migrate from southern overwintering areas within or south of the Potomac and Ohio river valleys to summer habitat throughout the eastern United States [6,7,8]. During the maternity season, eastern red bats normally day-roost in deciduous tree foliage singly or in small numbers [6,8]. Early summer parturition allows juveniles sufficient development time before engaging in migration and hibernation [9]. Species of the Family Vespertilionidae typically become volant within 3–4 weeks of birth, though the range is between 2 weeks and 2 months [10]. The timing of female’s hibernation emergence, pre-volancy weather conditions, food resources, and maternity roost conditions impact the timing of juvenile volancy: warmer roosts reduce thermoregulatory constraints on mothers and offspring, thus leading to shorter gestation periods and expedited juvenile development [9].

Acoustic monitoring is able to record commuting, foraging, and social bat echolocation call sequences, and these calls are often used as indirect measures of bat population size. Passive acoustic monitoring is often used in lieu of mist-netting, as it has a higher probability of detecting rare bat species than mist-netting surveys, which generally have low capture rates relative to bat activity [11,12]. Acoustic monitoring has been used to document bat species’ distributions and habitat associations, as well as within-night activity patterns relative to weather and white-nose syndrome (WNS) impacts [13,14,15,16,17,18]. However, the relationship between summer acoustic data and the timing of reproductive phases has not been well studied.

Because of the current importance of using acoustics to monitor bats, our objectives were to document bat reproductive timing in our mid-Atlantic study area and evaluate how reproductive condition data were related to acoustically recorded echolocation call data. We focused our study on big brown and eastern red bats, which are both prevalent within the region, providing sufficient capture and acoustic data to make this comparison. We predicted that peaks in acoustic activity would coincide with juvenile captures, reflecting population increases within the sampled area.

## 2. Materials and Methods

### 2.1. Study Area

**Figure 1:**
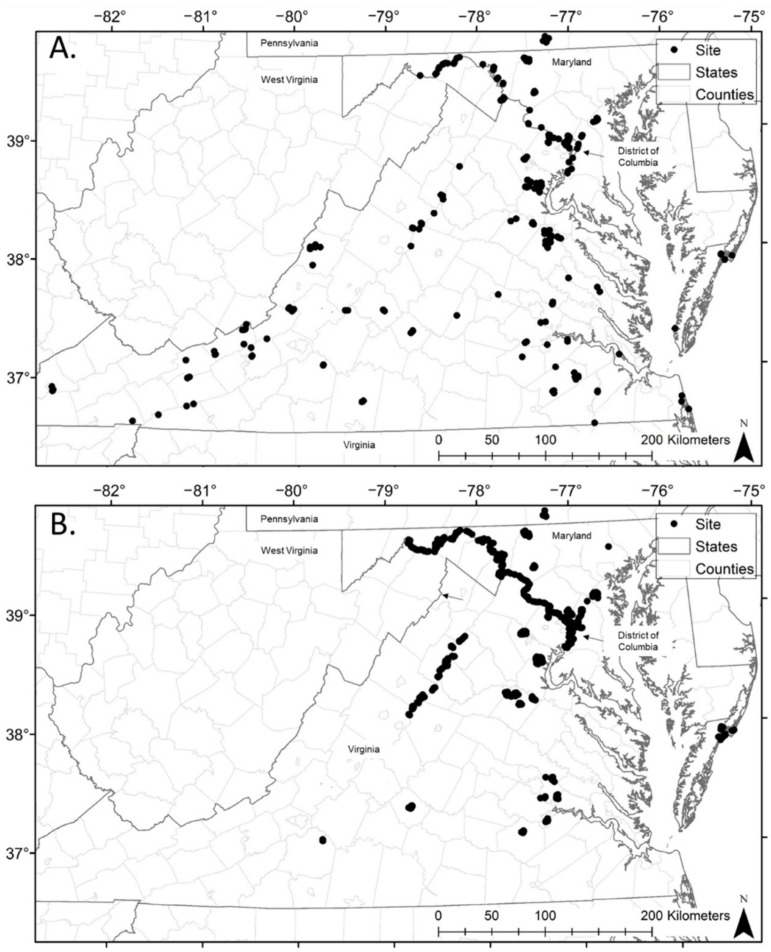
Mid-Atlantic sampling sites where (**A**) reproduction and (**B**) call data were collected between 2015 and 2018.

Our study area included federal (National Park Service, U.S. Forest Service, National Aeronautics and Space Administration and Department of Defense) and state (Virginia Department of Wildlife Resources, Virginia Department of Forestry and Virginia Department of Conservation and Recreation) lands within the Coastal Plain, Piedmont, Blue Ridge, and Ridge and Valley physiographic regions across the District of Columbia (D.C.), Maryland, Pennsylvania, Virginia, and West Virginia (Figure 1). Mean growing degree days (days over 10 °C) for 1980–2010 ranged between <67 in south-central Pennsylvania and western-most portions of Maryland, Virginia, and West Virginia, 67–100 for most of the study area, and 100–133 within a small southeastern portion of Virginia [19]. In general, temperatures were negatively related to increasing elevation and/or distance from the Atlantic Ocean. The western portions of the study area included large contiguous forests in mountainous areas with karst geology, and the northern and eastern areas contained more developed land and fragmented forest stands than did our more southern sites.

### 2.2. Methods

To collect reproductive condition data, we mist-netted between 2 May and 15 August 2015–2018 (Julian day 122–227; Figure 1). Because sampling occurred relative to the particular needs of several independent studies [20,21,22,23,24,25,26], sample location selection, the distribution of repeated surveys, and net effort varied greatly across the study area both within and between years. Regardless of study, we placed 38 mm low-bag single-, double-, and triple-high mist nets (Avinet Inc., Portland, ME, USA; Any use of trade, firm, or product names is for descriptive purposes only and does not imply endorsement by the U.S. Government) along possible flight corridors or near water and avoided netting in heavy rain or temperatures below 10 °C [14]. We captured and handled bats in accordance with Virginia Tech Institutional Animal Care and Use protocol 14-014 and 16-240. For each capture event (sample), we documented the species, age class (juvenile or adult), reproductive condition, and sex of each bat [27], and we calculated the proportion of pregnant, lactating, and juvenile big brown and eastern red bats captured per sampling event [28]. We determined age class through inspection of epiphyseal ossification and reproductive condition of females through palpation of the abdomen and condition of mammary glands [29,30]. Proportion of pregnant and lactating bats was calculated when adult female bats were captured, and proportion of juveniles was calculated when either adult or juvenile bats were captured. Nights with no captures of the species of interest were excluded from analyses of that species’ reproduction. We reviewed the spatial distribution of reproduction using simple plotting of dates with both geographic location and regression, and we determined that reproductive timing was likely not related to summer capture location (Supplemental Figure S1) [31].

We collected passive acoustic data from 10 April to 8 September (Julian days 100–250) in 2016–2018 (Figure 1). We used zero-crossing SongMeter (ZC, 2+, 3, and 4) and full-spectrum SongMeter 4 acoustic recorders (Wildlife Acoustics, Inc., Concord, MA, USA) to record echolocation calls from sunset to sunrise. Although most acoustic sampling sites were within forested (*n* = 555), edge (*n* = 81), and wetland (*n* = 176) habitat, we also surveyed open and developed sites (*n* = 82). Where possible, we placed the passive detector microphones at 3.66 m height on telescoping poles > 3 m from the bole of a tree (Loeb et al., 2015). To identify echolocation pulses to species, we used the Kaleidoscope Pro Bats of North America 4.2.0 classifier (Wildlife Acoustics, Inc., Concord, MA, USA) with U.S. Fish and Wildlife Service-sanctioned settings for acoustical monitoring and estimated file-level correct identification of 70% for both species [32,33]. Similar to the mist-netting capture data, acoustic sample site selection and sampling duration at each site within and between years were based on individual project criteria and, therefore, did not occur proportionally with net effort in all areas (Figure 1).

Similar to Francl et al. [28], we used Program R to calculate a locally estimated scatterplot smoothing (LOESS) line for the proportion of pregnant, lactating, and juvenile big brown and eastern red bats captured each Julian day [34]. We calculated the mean number of echolocation calls per Julian day for big brown and eastern red bats, and calculated the LOESS curve for both datasets. To determine peak maternity season activity, we identified the inflection point, or the Julian day, where the trend curve reached the highest asymptote, for each regression line. We used Julian day for our calculations, but report findings as calendar day in text.

## 3. Results

We captured bats between 30 April and 15 August (2015 = 7 May–7 August, 2016 = 8 May–12 August, 2017 = 30 April–10 August, 2018 = 5 May–15 August). We documented 1849 big brown bats and 824 eastern red bats. We documented adult female big brown bats on 219 nights (pregnant = 80 nights, lactating = 70 nights), and 340 adult female eastern red bats on 184 nights (pregnant = 51, lactating = 54 nights). We captured juvenile big brown bats on 82 nights and juvenile eastern red bats on 56 nights.

**Figure 2:**
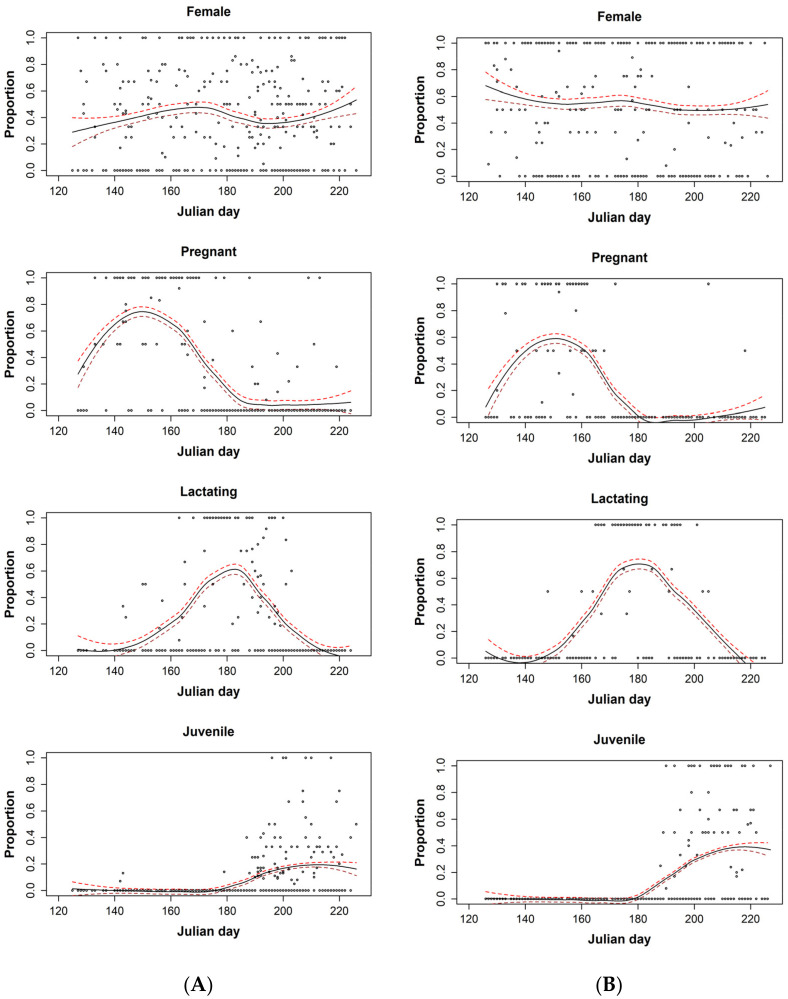
The samples (circles) and locally estimated scatterplot smoothing lines for the proportion of juvenile, pregnant, and lactating (**A**) big brown bats (*Eptesicus fuscus*; EPFU) and (**B**) eastern red bats (*Lasiurus borealis*; LABO). Mist-net sampling was conducted within the mid-Atlantic region between 2015 and 2018. Circles reflect each sample where the proportion of lactating female or juvenile bats was calculated.

The sex ratio among captures appeared to be relatively stable throughout the sampling period; although, the small increase in the proportion of female big brown bats captured later in the season may reflect an earlier departure by some males. We captured lactating big brown bats between 23 May and 22 July and lactating eastern red bats between 28 May and 23 July. We captured lactating big brown and eastern red bats in conjunction with pregnant and juvenile bats of both species; although, pregnant eastern red bat captures were rare after juvenile volancy (Figure 2). We documented the first big brown bat juvenile on 22 May (2015 = 28 June, 2016 = 5 July, 2017 = 7 July, 2018= 22 May), and the last pregnant female on 7 August. After the capture of a juvenile big brown bat on both 22 and 23 May within Maryland (Ft. George G. Meade), the next juvenile capture did not occur until 28 June. Starting 6 July, we documented juvenile big brown bats almost daily. We captured the first juvenile red bat on 7 July (2015 = 7 July, 2016 = 7 July, 2017 = 9 July, 2018 = 12 July), and the last pregnant female on 6 August, 13 nights after the previous capture of a pregnant bat. There were only two pregnant bats captured after 20 June.

We gathered acoustic data from 35,410 site-nights between 2016 and 2018 (Supplemental Figure S2). Individual detector sites were sampled between 1 and 232 nights (mean = 39.70 ± 45.93). Over the entire study, we identified 814,908 big brown and 194,994 eastern red bat call files on 20,885 and 12,983 nights, respectively. We noted relatively higher big brown bat activity (calls and captures) within the D.C. area and eastern red bat activity in the Blue Ridge and Ridge and Valley provinces. We observed lower levels of acoustic call activity during late-summer than in the spring.

**Figure 3:**
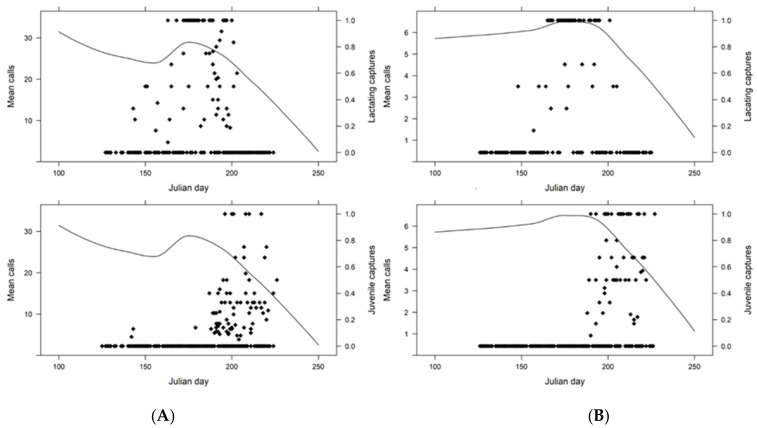
Proportion of juveniles and lactating females (black diamonds, right axis) within each sample and locally estimated scatterplot smoothing lines based on mean calls by Julian day (line, left axis) for (**A**) big brown bats (*Eptesicus fuscus*; EPFU) and (**B**) eastern red bats (*Lasiurus borealis*; LABO) within the mid-Atlantic region from 2015 to 2018. Black diamonds reflect each sample where the proportion of lactating female or juvenile bats was calculated.

The LOESS lines indicated that call declines began after 24 June for big brown bats with means dropping below 28 calls per night after 3 July (Figure 3). Though year and spatial distribution impacted observed patterns (Supplemental Figure S3) [31], the relative relationships of the asymptotes to reproduction were similar. Eastern red bat calls exhibited a summer asymptote on 23 June, and declined below a mean six calls per night on 17 July. Mean calls for both species plateaued mid-summer, and steep declines in mean calls per night corresponded with a decrease in the proportion of pregnant bats, an increase in the proportion of lactating bats, and before the emergence of most juvenile bats (Figure 2 and Figure 3). After eliminating two outlier juvenile big brown bat captures (22–23 May), the first juvenile capture did not occur until after the summer acoustic inflection point, and we did not routinely capture juveniles until 12 nights after the acoustic inflection point. The difference between the acoustic inflection point and the first juvenile eastern red bat capture was 14 nights. The big brown and eastern red bat capture inflection points were similar (pregnant = 30 and 31 May, respectively; lactating = 2 July and 29 June, respectively; juveniles = 31 July and 7 August, respectively; and acoustic activity = 24 and 23 June, respectively). The acoustic inflection point for the entire study area was closest to the lactation inflection point (8-night difference for big brown bats and 6-night difference for eastern red bats) and furthest from the juvenile inflection points (37-night difference for big brown bats; 45-night difference for eastern red bats).

## 4. Discussion

The differences between lactation and juvenile inflection points (big brown bats = 29 days, eastern red bats = 38 days) were comparable to periods between parturition and volancy documented previously for both species [4,8]. The lactation inflection point for big brown bats was similar to that observed by Francl et al. [28] in West Virginia, and the juvenile inflection point was 8 nights earlier. In southwestern Virginia, Timpone et al. [35] documented juvenile big brown bats slightly earlier than our observations. Our eastern red bat reproduction inflection points were very close to those found post-WNS in West Virginia [28]. We captured a pregnant eastern red bat 26 nights and a juvenile eastern red bat 13 nights later than Timpone et al. [35], respectively. The timing of initial captures of lactating eastern red bats was similar to those reported by Timpone et al. [35]; although, our captures continued beyond their reported time frame. Some differences in the earliest and latest captures between studies are attributable to sample size and sampling extent. The likelihood of catching bats at a particular reproductive condition is related to the proportion of bats currently in that condition. Therefore, greater netting efforts may be needed to fully document reproductive ranges outside of peaks in the cycles [31].

Colder temperatures can increase gestation length due to a decrease in prey availability and/or an increase in thermoregulatory energy requirements [9,36]. Therefore, results from a small number of colonies in similar environmental conditions will likely yield a smaller range of parturition and volancy dates than results from a greater number of colonies and/or colonies with more diverse habitat and roosting conditions. In contrast, solitary-roosting bats, such as eastern red bats, may experience highly-variable individual roosting conditions within a similar area.

The large temporal range of big brown bat pregnancies indicates high plasticity in big brown bat reproduction regardless of location. The two earliest juveniles captured were in a suburban portion of Maryland; however, these early captures were not representative of the reproductive timing of other urban big brown bats. These juveniles may have been attributable to the previous year, as has been rarely observed [37].

Our results indicate that for big brown bats, and to a lesser extent eastern red bats, there is a period of overlap in which pregnant, lactating, and some juvenile bats are concurrently volant, thus potentially contributing to acoustic activity peaks. However, our data indicate that peak acoustic activity occurred before most juvenile captures. The LOESS line peaks in acoustic activity corresponded more with the highest capture rates of pregnant and lactating females in both species.

These results indicate a contradiction of assumptions of past research that has attributed peaks in acoustic activity to juvenile volancy, i.e., a greater abundance of bats flying, and indicative of successful reproduction [13,17]. The highest activity may be due to energetic demands, as females approaching parturition or currently lactating engage in longer and/or more frequent foraging bouts [9,38,39,40,41,42,43]. In Manitoba, Canada, female hoary bats (*Lasiurus cinereus*) had longer foraging bouts when they had two offspring versus one [38], and both eastern red bats and big brown bats often have more than one offspring [4,8]. Maternity activity and/or colonies begin to disaggregate with juvenile volancy, beginning migration or mating activities before hibernation [9]. Correspondingly, we observed exponential decreases in acoustic activity as the proportions of lactating bats decreased. These decreases occurred within periods that both the North American Bat Monitoring Program and USFWS Indiana bat survey guidance suggest as appropriate for listed eastern bats’ maternity-season surveys [14,18]. For a balanced spatial–temporal sampling approach, we suggest that researchers review previous acoustic and reproduction studies in their study area to determine when fall migration may occur. If no such data exist, collecting data from multiple periods within the maternity season and evaluating post hoc the beginning of call declines might indicate the onset of fall migration. The precipitous declines in both adult captures and acoustic recordings likely indicate the beginning of migration from big brown and eastern red bats commencing shortly after juvenile volancy. This conclusion is supported by Walters et al. [44], who observed adult red bats in Indiana vacating summer habitat prior to juveniles.

We observed some of the highest big brown acoustic activity in the spring (prior to 20 April), which we attribute to a combination of migration, exploratory movements, and increased foraging activity of prenatal females [45]. Muthersbaugh et al. [46] also observed acoustic activity decreasing at a consistent rate between early March and late April in the Virginia Appalachian Mountains, supporting our interpretation that declining activity in May was related to spring migration. Additionally, our acoustic sampling likely included fall migration given sharp declines in mean calls. This result is similar to Muthersbaugh et al. [46], where Julian day did not predict eastern red bat acoustic activity from September through mid-November, perhaps because bats had largely ceased migration by that period. Our call asymptote and late-summer declines are similar to those patterns observed in eastern red bats in areas with viable roosting habitats along the nearby Delmarva Peninsula [47]. Further analysis to determine large-scale patterns and habitat use of eastern red and big brown bats during these migration periods is needed.

## 5. Conclusions

Acoustic sampling provides a valuable method of indirectly measuring bat activity, but it has not been reliably associated with population demographics. Our documentation of the relationship between peak maternity season acoustic activity, pregnancy, and lactation, rather than juvenile volancy, provides managers with new information on how to interpret bat acoustic results and assess temporal sampling biases. Because acoustic activity appears related to reproduction and migration patterns, summer-long acoustic recordings could be used to identify reproductive patterns within a discrete area. Nonetheless, additional research would be an aid in interpreting acoustic data from different short-term sampling periods, or comparing acoustic data collected over a broad spatial and temporal scale may result in community, habitat association, or reproduction timing mischaracterizations. If our observed relationships between acoustic and capture data for these common species are similar to those for federally listed bats, then the current federal sampling guidelines probably encompass migration periods within the mid-Atlantic. As a result, sampling during these periods potentially could be misleading with higher false absences due to migration away from a maternity area or false indications of maternity colony presence based on migration through an area.

## Data Availability

The data presented in this study are available on request from the corresponding author. The data are not publicly available as related analyses are in progress.

## Supplementary Materials

The following are available online at https://www.mdpi.com/article/10.3390/d14050319/s1, Figure S1:The Julian days on which juvenile (A) big brown bats (*Eptesicus fuscus*) and (B) eastern red bats (*Lasiurus borealis*) were captured by site latitude and longitude, Figure S2: Histogram with the number of acoustic samples collected between Julian day 100 and 250 within the mid-Atlantic region from 2015 – 2018, Figure S3: Locally estimated scatterplot smoothing lines based on mean calls by Julian day for big brown bats (*Eptesicus fuscus*; EPFU) and eastern red bats (*Lasiurus borealis*; LABO) within the mid-Atlantic region for each year 2016 – 2018 and for the entire period.
