# Supplementary material for: Mid-Atlantic Big Brown and Eastern Red Bats: Relationships between Acoustic Activity and Reproductive Phenology
Source: Diversity (Basel). 2022 Apr 21;14(5):319. doi: 10.3390/d14050319 (PMC9185541; doi:10.3390/d14050319)
Supplement: Supplementary file 1 [file diversity-14-00319-s001.zip › diversity-1599000-supplementary.pdf]

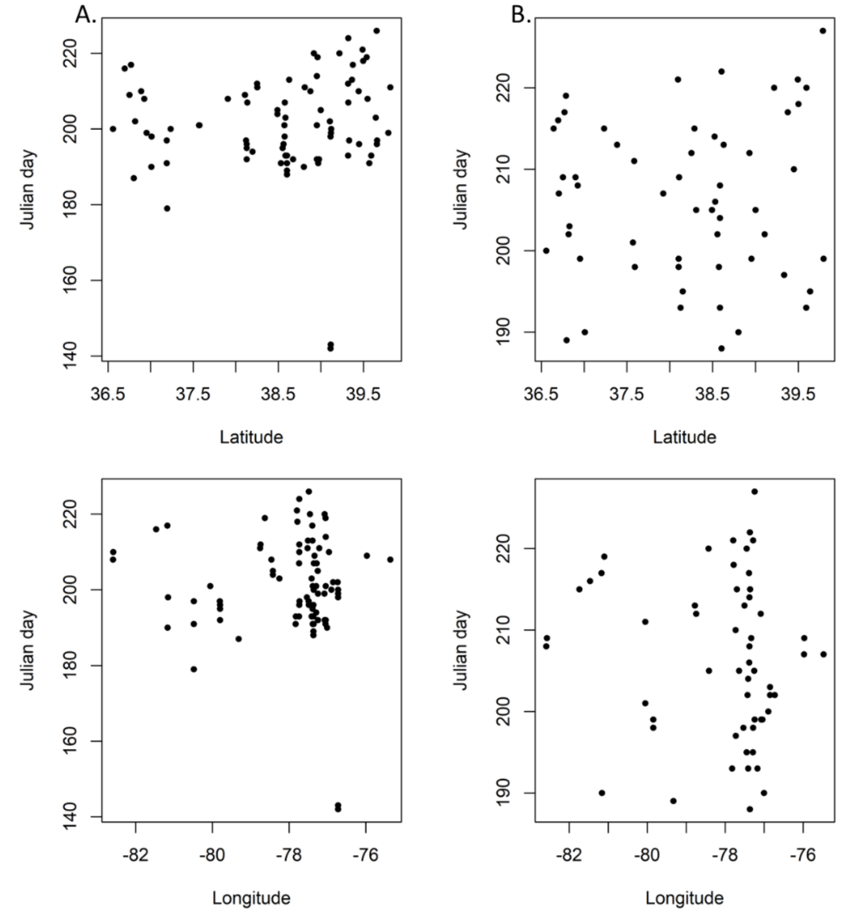

Figure S1. The Julian days on which juvenile (**A**) big brown bats (*Eptesicus fuscus*) and (**B**) eastern red bats (*Lasiurus borealis*) were captured by site latitude and longitude. Capture data are from mist-net sampling nights (2015 – 2018) conducted throughout the mid-Atlantic.

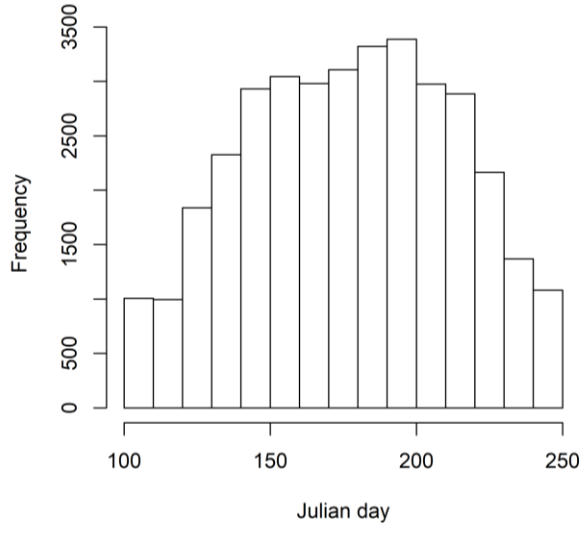

Figure S2. Histogram with the number of acoustic samples collected between Julian day 100 and 250 within the mid-Atlantic region from 2015 – 2018.

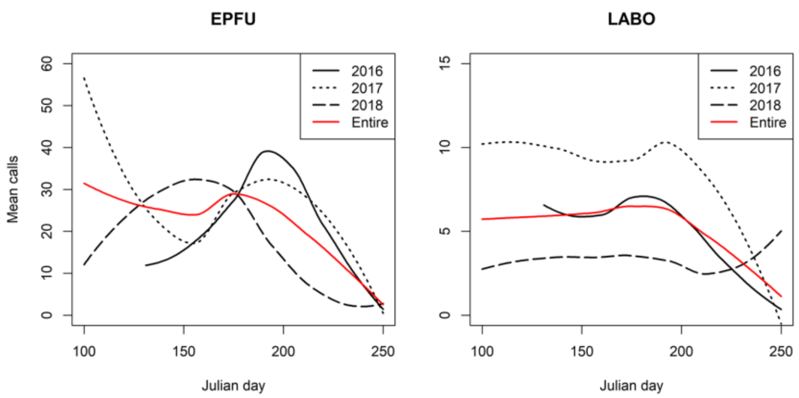

Figure S3. Locally estimated scatterplot smoothing lines based on mean calls by Julian day for big brown bats (*Eptesicus fuscus*; EPFU) and eastern red bats (*Lasiurus borealis*; LABO) within the mid-Atlantic region for each year 2016 – 2018 and for the entire period.
